# Non-Pharmacological Treatments of Cognitive Impairment in Multiple Sclerosis: A Review

**DOI:** 10.3390/neurosci3030034

**Published:** 2022-08-22

**Authors:** Michela Bossa, Nicola Manocchio, Ornella Argento

**Affiliations:** 1 Behavioral Neuropsychology Laboratory, I.R.C.C.S. “Santa Lucia” Foundation, 00179 Rome, Italy; 2 Physical and Rehabilitation Medicine, University of Rome “Tor Vergata”, 00133 Rome, Italy

**Keywords:** multiple sclerosis, cognitive impairment, treatments

## Abstract

Cognitive impairment (CI) represents a common symptom in patients suffering from multiple sclerosis (MS), which can affect every stage of the disease course. Recent studies seem to support cognitive rehabilitation (CR) for minimizing the CI consequences. We reviewed the currently available evidence on the non-pharmacological approaches to CI, with the aim of giving an overview of the treatments used worldwide, from the traditional methods to the most recent techniques. A search of the literature was conducted on PubMed (articles in English performed in the last five years on humans). A total of 37 articles met our eligibility criteria after screening titles, abstracts and full-text and were divided into three main groups: in-presence interventions; studies performed via tele-rehabilitation and miscellaneous. Despite the great heterogeneity of the intervention and assessment methods, the evidence suggests that a non-pharmacological approach can improve MS-related CI. Cognitive rehabilitation seems effective and well established, as well as the use of computerized CR having the benefit of being even more appealing. Limited conclusions can be drawn on group CR due to the small number of studies focused on this kind of intervention. Some of the innovative approaches (virtual reality, EEG-based neurofeedback, brain stimulation, exercise, diet modification) may play a role in future studies and should be deeply explored.

## 1. Introduction

Cognitive impairment (CI) occurs in 43–70% of patients with multiple sclerosis (MS) [1] and cognition is compromised in MS patients in every stage of the disease.

In particular, the most prevalent deficits observed are slowed information processing speed, inefficient learning and long-term memory (ability to learn new information and to recall that information at a later time point), but also impairment in attention, working memory (information that can be held in mind and used in the execution of cognitive tasks), verbal fluency, executive and visuospatial functions [2].

The patients with CI had greater difficulties when performing routine tasks than those who had a purely physical disability, leading to a reduced participation in social activities and/or employment, and a reduced quality of life. To date, there is insufficient evidence to support the use of a pharmacological intervention with disease-modifying therapies in order to improve cognitive function [2]. Moreover, in the last few years the effects on cognition of other pharmacological interventions were also studied, such as acetylcholinesterase inhibitors (which can be administered as these drugs increase the levels of acetylcholine, a neurotransmitter involved in learning and memory) and amphetamines (which can be used to treat processing speed abnormalities) with low level [3] or without statistical significance [4].

Recent reviews were focused on the role of cognitive rehabilitation (CR) in MS patients. Taking into account the Cochrane reviews, systematic reviews and meta-analyses performed in the 2011–2020 period [5] and other randomized clinical trials (RCTs) performed in the 2013–2021 period [6], and those focused only on memory rehabilitation [7], the effectiveness of CR seems to be supported. However, not all of the CR studies reported in the literature were considered in these reviews, as well as other studies about innovative therapeutic approaches, efficacious in other neurological conditions.

Therefore, the purpose of this report was to provide current knowledge, based on the newest publications, regarding all of the non-pharmacological treatments of CI in MS, spanning not only cognitive rehabilitation but also other non-pharmacological approaches.

## 2. Materials and Methods

A search of the literature was conducted using the following terms, both alone and in combination, on the PubMed database: multiple sclerosis (MS); cognition; cognitive impairment; cognitive dysfunction(s); cognitive disorder(s); attention; memory; information processing speed; working memory; executive functions; visual–spatial functions; non-pharmacological treatment; non-pharmacological therapies; rehabilitation.

The search was limited to the studies published in English in the last five years on humans. Two reviewers (MB and NM) independently screened the titles and abstracts for the full manuscript review and third-party consensus was used when needed.

The articles were excluded if: (1) the article was not peer reviewed (e.g., book chapters); (2) the publication was a review article on the research topic; (3) the study was not an intervention; (4) the publication was a case report without empirical data to evaluate the outcomes; (5) a total of >50% of the participants did not have MS; (6) the study was focused on a sample of subjects with pediatric-onset MS; (7) the study was focused on MRI techniques without behavioral data; (8) the study was focused on cognitive assessment or risk factors of cognitive impairment or other aspects and disorders present in MS patients; (9) the intervention was not targeting a cognitive domain; (10) the intervention under investigation was pharmacologic; or (11) the intervention under investigation was cognitive behavioral therapy for the treatment of psychological symptoms.

Following the initial abstract evaluation, the corresponding full-text manuscript was retrieved. The same reviewers (NM and MB) assessed each full-text article for eligibility using the standardized criteria as described above, and extracted the data regarding: sample size (number of patients in each group, missing participants); study design; interventions; outcomes; and what type of outcome was used to document efficacy and generalization (cognitive and/or MRI results). The extracted data were transcribed into standardized data collection sheets.

The cited references of the included articles were screened for potential inclusion.

This review followed the Preferred Reporting Items for Systematic Reviews and Meta-analyses (PRISMA) guidelines.

The report was written by MB and NM and reviewed by OA.

## 3. Results

**Figure 1:**
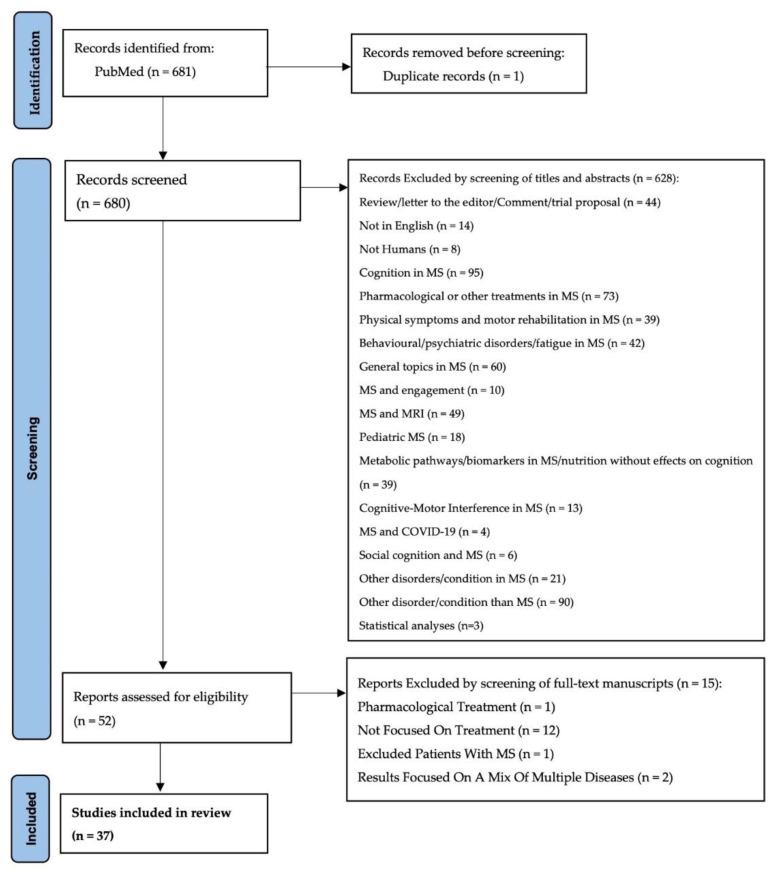
Flowchart of the study selection process.

Figure 1 shows the study selection process.

The initial search yielded 681 citations with one article duplicate, and then the following 680 records were screened in two-step revision:

1. By screening titles and abstracts;
2. By screening of full-text manuscripts.

In the first step, 628 articles were excluded based on the aforementioned criteria.

In the second step, 15 additional articles were excluded if: (1) the study was not an intervention, (2) the intervention under investigation was pharmacologic, (3) the study population was composed of multiple disorder or MS patients were excluded during the study.

In conclusion, 37 articles met our eligibility criteria.

Table 1 shows the different type of non-pharmacological treatments we considered in this review.

### 3.1. Cognitive Rehabilitation

CR has gained attention in recent years, and several studies have investigated the efficacy of CR in the treatment of CI in MS patients. The aim of CR is the learning and the development of cognitive abilities by training specific tasks, performed with different approaches and techniques: compensatory strategies; internal and external memory aids; making use of mental reviewing methods; error-free learning; solutions to focus attention and concentration; methods of coping with memory problems [8,9]; computer-assisted programs; textbook exercises; story memory technique; use of diaries; calendars; notebooks and lists; repetition effect; multicomponent cognitive rehabilitation; and use of videogames [45].

These approaches were generally performed as individual or therapist-guided training, but in the last few years group CR has also shown efficacy (group CR, GCR: education program, problem-based learning, and home exercise assignments) in terms of memory and executive function [20], but also in subjective reports of memory impairments assessed by the Everyday Memory Questionnaire [21].

The treatment protocols often focus on one domain of cognition, as memory: both the working and the everyday memory improved after traditional CR (one-hour sessions on a weekly basis for eight weeks), as reported in two articles by Mousavi et al. [8,9], while after the adaptive n-back working memory training by Turtola [10] an enhancement of attention and cognitive control was seen during untrained tasks in a sample of people with MS. Another aspect, the Autobiographical Memory (AM) part of the retrograde memory, was treated in a tailor-made Mental Visual Imagery (MVI)-based facilitation program with mental visualization exercises of increasing difficulty [11]. In this study, only the experimental group showed a significant AM improvement and functional/structural changes such as: increased reliance on brain regions sustaining self-referential process; decrease of those reflecting a research process; better use of neural pathways in the regions sustaining MVI.

Therefore, the other studies have focused on multimodal treatment paradigms designed to target several domains of cognition, either simultaneously or consecutively, because the patients’ behavior and the consciousness of their disease state are two important factors that can affect cognition.

In 2020, Brissart et al. [12] conducted a study using the French ProCog-SEP (Program Cognitif pour Sclérose En Plaques) program, based on psychoeducational advice and cognitive exercises in a textbook. A total of 64 patients were assigned to the experimental group and performed 13 group sessions over 6 months, showing better results in working memory and verbal learning in comparison with the control group.

The Modified Story Memory Technique (mSMT), a treatment protocol that teaches patients to utilize contextualization and visual imagery strategies to facilitate learning and then to apply them to real-world settings, was used by Chiaravalloti et al. [13] in a progressive multiple sclerosis sample with an improvement in new learning, but also an increase in the awareness of cognitive deficits.

The Strategy-based Techniques to Enhance Memory (STEM) is a new treatment paradigm aimed at teaching patients the principles of self-generation, spaced learning and retrieval practice, and how to apply these techniques in daily life. In a recent study [14], a medium-large effect size was noted on the score of the objective test of learning ability (California Verbal Learning Test—second edition, CVLT-II), indicating that a statistical significance may be observed with a larger sample size.

In addition, Goverover et al. [15] designed a protocol based on behavioral intervention, teaching self-generation techniques and metacognitive strategies to increase learning and memory abilities. A total of 35 patients were randomized into an experimental group (19) and a control group (16), who performed less complex learning and remembering activities. Both groups underwent six individual sessions, of 60 min each. The treatment group improved in learning memory, self-regulation and metacognition.

Pineau et al. [16] explored how a psycho-educational program they developed (the ADACOG program: adaptive program for cognitive and emotional deficits) affects CI. ADACOG focuses on cognitive and emotional dysfunctions in MS and provides strategies to cope with them; patients, divided into small groups, are required to attend three modules (1: cognitive dysfunctions; 2: emotional symptoms; 3: rehabilitation and coping strategies) divided into two hour sessions every two weeks. Forty-five patients with self-reported and objective CI were enrolled in a treatment group (24 subjects) and a control group (21 subjects). Both of the groups were asked to complete several questionnaires, among which was the Multiple Sclerosis Neuropsychological Screening Questionnaire: after the treatment, the experimental group showed less self-reported cognitive deficits.

In another neuropsychology-based study, Stimmel et al. [17] explored the use of additive interventions (in-person feedback and care-coordinator phone calls) on women with MS and CI. Sixteen employed patients were allocated to an experimental group and fourteen in a “standard care” group (who received feedback and recommendations via phone). The authors found that the protocol was feasible and acceptable, but no significant difference in relation to cognition was noted pre- and post-intervention between the two groups.

Cognitive Occupation-Based Programme for People with Multiple Sclerosis (COB-MS) was developed, in order to facilitate people with MS to engage more effectively in daily life activities and cognitive tasks, that they find difficult as a result of their impairment [18]. This program (eight sessions: two individual, six group-based), was focused on education, remediation and adaption of the patients by the use of compensatory strategies and routines and learning new techniques. Thanks to this approach, the patients improved in daily life outcome measures and most of the cognitive outcome measures.

Finally, in the case-control study by Impellizzeri et al. [19], the traditional CR was compared with an integrative approach, composed of CR and neurologic music therapy (NMT). In recent years, musical training was widely applied in the neurological rehabilitation context, due to the role of music in neuroplasticity, and is composed of a variety of interventions. Impellizzeri et al. used the Associative Mood and Memory Training and the Music in Psychosocial Training and Counseling for 8 weeks and found that NMT could be considered as a complementary approach to enhance CR: the experimental group showed an improvement greater than the control group in the selective reminding test-long term storage, long term retrieval and delayed recall of the 10/36 spatial recall test. A similar outcome was found regarding emotional status, mood, motivation and quality of life.

#### Computerized Cognitive Rehabilitation (CCR)

In the most recent years, great attention has been given to the use of computer-assisted Cognitive Rehabilitation. The spread of computers, tablets and smartphones has made people more confident with the use of technology. At the same time, technological advances have allowed more innovative solutions. Accordingly, we found several articles about this topic.

Different programs can be used in CCR with promising results. In 2018 Arsoy et al. [22] conducted a trial based on the NOROSOFT Mental Exercise Program (focused on five domains: attention, memory, reasoning, visual, and verbal tasks); 10 of 21 Benign-MS patients (BMS), randomly assigned to the CCR, improved in sustained attention, information processing speed, verbal fluency, categorical reasoning, and executive functions, compared to the 38 controls.

Similar findings were gathered by the MAPSS-MS (Memory, Attention, Problem Solving Skills in MS) intervention in a multi-site trial [23]. This intervention included both group sessions and a computer protocol. The first one (2 h/week for 8 weeks) aimed to develop relevant compensatory strategies for cognitive deficits through verbal persuasion, performance accomplishment of new behaviors and role modeling. The home-based computer training program (45 min three times per week) was composed of a series of cognitive tasks/games: Birdwatching; Word Bubbles; Monster Garden; By the Rules; Penguin Pursuit. The comparison group received instead the usual care plus freely available computer games. The experimental group improved in the CVLT Delayed Score, 3 second-version Paced Auditory Serial Addition Task (PASAT 3′), Controlled Oral Word Association Test (COWAT) and PROMIS (^®^) Cognitive Abilities scale.

Blair et al. [24] designed a pilot RCT based on the Cogmed Working Memory Training (CWMT), a five-week computer-assisted training program supported by weekly meetings with a coach. A total of 15 MS patients assigned to the CWMT group showed improvements in attention, working memory and mood.

ERICA is a piece of Italian software, which consists of personalized PC-exercises involving five domains: attention process; memory abilities; spatial cognition; verbal and nonverbal executive functions. De Luca et al. [25] used this program on 20 patients with MS and mild to moderate cognitive impairment, showing significant effects in memory, attention, and processing speed compared to the controls.

COGNI-Track is another piece of Italian software specifically developed for the rehabilitation of MS patients: it is a tablet app that allows people to train their memory and is able to personalize treatment, adapting the exercises’ difficulty according to a patient’s performance. Bonzano et al. [26] used COGNI-Track on 18 patients who performed an 8-week home-based training for working memory, with five 30-min sessions per week. After the training, the patients’ performance in PASAT significantly increased. A strength of Bonzano’s study was the evaluation of brain activation via functional Magnetic Resonance Imaging (fMRI): patients were scanned while performing the Paced Visual Serial Addition Test (PVSAT) and, after the training, the brain activation map was very similar to that of the healthy subjects.

BrainHQ is a validated online interactive brain training software used for restorative CR in people with MS. The research version of BrainHQ was used for the adaptive cognitive remediation (ACR) program: a remotely supervised cognitive training program developed via telerehabilitation, which has demonstrated efficacy in an active-placebo-controlled study, improving the cognition composite scores relative to the placebo [27].

In addition, Fuchs et al. [28] applied this software in a 12-week at-home study with 51 patients and found significant improvements in attention, processing speed and working memory evaluated with the Symbol Digit Modality Test (SDMT) with better results in the people with a relapsing–remitting (RR-MS) disease course.

Vilou et al. [29] used the same software with 23 patients with RR-MS in a 6-week cognitive rehabilitation intervention (two 40-min sessions per week) and compared the results with a group of 24 patients with the same type of disease. Vilou’s results contrasted with Fuchs’ as far as the SDMT is concerned, since the former’s study was not able to find any statistically significant improvement; on the other hand, the verbal and nonverbal episodic memory, reading speed, visual attention, verbal memory and visual attention significantly improved.

Darestani et al. [30] found out that RehaCom, a piece of software with acknowledged capabilities of improving cognitive functions, could improve verbal fluency, verbal learning and memory in 30 MS patients who performed 10 1-h training sessions in a 5-week program, compared to the controls.

A big role in CCR is nowadays played by videogames (VG). In rehabilitation, it is possible to talk about “serious” games when referring to VG developed with the purpose to train specific skills and to monitor difficulty progression; another frequently used term is “exergaming” (or “exergames”): this describes the combination of VG and exercise. VG make patients feel more involved and create a motivating environment; thus, they can increase compliance and performance. It is also possible to use them to perform dual-task rehabilitation, in which both the cognitive and motor skills are trained. Ozdogar et al. [31] evaluated the effects of a once-a-week, 8-weeks program of exergaming, performed with a home game console, on upper extremity and cognitive function in 21 RR-MS or secondary progressive (SP-MS) patients, matched with 19 controls performing conventional rehabilitation (balance, arm and core stability exercises) and 20 persons in a control group. The experimental group showed significant improvements in finger dexterity (Nine-Hole Peg Test), and in cognitive functions measured with the SDMT; working, visual and verbal memory were also better after the training protocol (while only the last two improved in the conventional rehabilitation group).

Bove et al. [32] conducted an in-home, tablet-based videogame-like protocol for 23 MS patients matched with a group of 21 people, who performed an active tablet-based placebo control. The software used for the study involves the patient in two simultaneous tasks (sensory and motor) and is designed to stimulate the frontal neural networks; it is also able to automatically adapt both in real time and between sessions according to the performance. The authors found a statistically significant increase in SDMT for both the treatment and the control groups, with a better result for the first group. The performance in the PASAT test also significantly improved in the experimental group. The authors evaluated the persistence of the findings and noted that it was higher in the experimental group than in the control group.

A different kind of CCR is Virtual Reality (VR), a simulated reality that uses technologic tools to create artificial environments, resembling reality itself, with which the patient can interact.

In a study by Leonardi et al. [33], 15 RR-MS patients were assigned to a VR rehabilitation protocol and matched with 15 controls receiving conventional cognitive rehabilitation (exercises with pencil and paper). The two groups performed three sessions a week for 8 weeks and in both groups an improvement was found in the visuospatial skills. However, a better cognitive result was achieved in the experimental group who showed a significant improvement in learning ability, short-term verbal memory and lexical access ability.

Maggio et al. [34] recently published a study evaluating the effects of a semi-immersive VR rehabilitation protocol on 30 RR-MS and SP-MS patients, matched with an equal number, conventional rehabilitation-control group (face-to-face approach between the patient and the therapist in individual sessions). Both of the groups underwent the same amount of CR (three sessions per week, each lasting 60 min, for 8 weeks, for a total of 24 sessions), but only the experimental group received the VR protocol. The authors found significant improvements in the visual perception, visuo-spatial abilities, short term visual memory, working memory, executive functions, information processing speed and sustained attention (2 second-version PASAT, PASAT 2′) in the experimental group.

### 3.2. Exercise Training (ET)/Physical Activity (PA)

In the literature, ET (considered as a type of planned, structured and repetitive physical activity, executed to improve the health-related aspects of fitness) and PA (considered as bodily movement and energy expenditure) were included in the rehabilitation paradigms for restoring motor function in mild to moderate MS.

Furthermore, there is emerging interest in investigating whether exercise also has an impact on cognition, due to recent neuroimaging findings, which have revealed that some areas in the thalamus and the hippocampus responsible for cognitive functions were also associated with physical performance in MS patients [46,47]. In more detail, the hippocampal lesions and atrophy were associated with MS-related memory impairment, while the thalamic atrophy was associated with slowed cognitive processing speed, impairment in learning and memory, verbal fluency and spatial perception, but also with compromised ambulation in MS patients [48].

In a recent review by Motl and Sandroff [47], exercise-related increases in the hippocampal volume and the hippocampal resting-state functional connectivity were described, in the resting state functional connectivity between the thalamus and pre-frontal cortex and in the verbal and non-verbal memory performances. Thus, the exercise was considered as a countermeasure to the declining Central Nervous System function in a patient with MS, due to its influence on both brain structure and cognitive function.

This widely confirms a previous study [49], in which the authors developed a rationale for considering the efficacy of exercise training in MS, comparing the positive evidence presented in gerontology. In fact, aerobic fitness, PA and ET were associated with better cognitive function in older adults, and ET has had comparable effects on mobility and quality of life outcomes in older adults and persons with MS.

According to a previous study [50], revealing that a combined exercise training increased the serum levels of brain-derived neurotrophic factor (a neurotrophin that plays a role in preventing neurodegeneration), the same research group have demonstrated [38] that a combined exercise training (aerobic and Pilates training; three sessions/week for 8 weeks) improves the long-term verbal memory, visuospatial memory, verbal fluency and information-processing speed.

Barry et al. [35] demonstrated that a short (two times/week for 8 weeks; 30-min exercise session) routine cycle ergometry training was associated with an improvement in attention, executive function/cognitive flexibility and visuospatial memory.

PA might also be performed at home with the supervision of the therapist, as the community-located “Start-to-Run” Program, which lead to an improvement in visuospatial functions [39] or the community-based program via the WalkWithMe application, with a small improvement in information processing speed [40].

MS patients can have serious motor deficits: to enhance their rehabilitative possibilities some wearable devices can be applied, such as an exoskeleton. Androwis et al. [36] designed an RCT to evaluate the effects of a 4-week gait-training program with a Robotic Exoskeleton, comparing the results with a control group that performed Conventional Gait Therapy. Two people with RR-MS were assigned to the experimental group and the other two to the control group; both of the groups performed their training in a gym, supervised by a physiotherapist. The patients who used the RE increased their motor abilities, but also processing speed, giving credit to the idea that motor functions influence cognition.

Langeskov-Christensen et al. [37] tried an approach with progressive aerobic exercise to increase cognitive abilities in MS patients. A total of 43 people performed 24 weeks of aerobic exercises, while a control group kept the previous lifestyle without changing physical activity levels. The authors found no differences after the training period between the two groups; they then assessed a sub-group of cognitive impaired patients: this population showed a relevant increase in processing speed through improvement in the SDMT scores. Thus, Langeskov-Christensen’s study is in contrast with the other findings on the effect of exercise training on cognition in not-impaired populations, but confirms its usefulness on CI.

### 3.3. Non-Invasive Brain Stimulation and Brain Modulation

Two appealing therapeutic options in the management of cognitive disorders in MS are the non-invasive brain stimulation (NIBS) and the brain modulation via EEG-based neurofeedback (NF) training: in fact, by facilitating or inhibiting neuronal activity, these treatments play an important role in neuromodulation.

In particular, we found two articles on the transcranial Direct Current Stimulation (tDCS), also remotely supervised (RS-tDCS).

An improvement in reasoning and executive functions was demonstrated in the first study with a multi-session tDCS protocol [41]. Also in the other study focused on the RS-tDCS paired with a cognitive training (CT) program, it was demonstrated an improvement in complex attention and response variability composites compared to the CT only [42]. 

In the NF training by Pinter et al. [43], the brain activity was recorded and fed back to the subjects in real-time, in such a way that the patients modulated their own cerebral activity. There was cognitive improvement, and also increased white matter integrity and functional connectivity in the brain regions associated with self-regulation, motor, and cognitive function.

All of the studies previously mentioned are summarized in Table 2 (in-presence studies) and in Table 3 (studies performed via telerehabilitation).

### 3.4. Other Interventions

In addition to the approaches previously reported, there is another—unconventional in a way—therapy that can be applied to treat MS and that has an impact on CI (Table 4): the dietary changes considered in the work by Petrou et al. [44].

The authors used GranaGard, a food supplement made of pomegranate seed oil that claims to prevent neuronal death. A total of 30 patients were equally divided into two groups: the first received GranaGard for three months and placebo for the next three; the second group did the opposite; both received the food supplement for six extra months after the first. The verbal learning (assessed via CVLT-II) increased after 3-months treatment with GranaGard in both of the groups; in the first group, who took the product for the first period, the improvement seemed to last longer, even in the placebo phase. No difference was found in processing speed (via SDMT).

## 4. Conclusions

Given the profound impact of CI on people with MS, a therapeutic approach is mandatory: this can be performed with a single type of treatment (especially if the results of only one cognitive domain are compromised) or by combining several types of treatment.

The available evidence suggests that CR can ameliorate MS-related cognitive impairment. Earlier studies were concentrated on memory; more recent studies shifted their focus to treatment of executive function, sustained attention and information processing speed, that are important aspects of real-life situations.

Due to the small number of studies focused on group cognitive rehabilitation in our search [20,21], limited conclusions can be drawn from it: it is relevant to establish the efficacy of this approach because it requires fewer resources, being low-cost and low-risk (in comparison with the pharmacological therapies), and could increase patients’ compliance.

CCR seems to be as efficacious as CR, and more appealing, especially for those programs with an adaptive training approach in which the difficulty level of the training is adjusted according to the trainee’s performance. This aspect could also stimulate patients’ adherence to treatment.

The emerging approaches counted in this report (VR, EEG-based neurofeedback, NIBS, NMT, ET) play a role in the different neural aspects (neuromodulation, microstructural WM integrity, increased functional connectivity and brain activation), suggesting a training-related neuroplasticity and leading to an improvement in cognition. For this reason, another important aspect that we want to emphasize concerns the outcomes taken into consideration in the selected articles: in fact, in the past, the main core of the outcome evaluation was the cognitive functioning via neuropsychological tests. Nowadays, according to the growing interest in functional connectivity, outcome evaluation also comprises imaging parameters, especially those which are task-related.

Moreover, many of the studies reported in this review are home-based, combining CCR, PA, RS-tDCS and EEG neurofeedback, with the social and economic impact. In fact, the possibility of treating patients at their home is useful both in situations such as pandemic conditions (when people are forced to limit their access to clinical centers) and in situations in which patients have no possibility of reaching rehabilitation centers. The economic advantage of telerehabilitation consists of the reduction in the impact on both the clinical facilities and patients. In particular, they do not require the patient to spend time and money to travel to and from the rehabilitation facilities.

The evidence of the efficacy of telerehabilitation is still limited, but the results that we have found encourage further studies in this field.

Finally, miscellaneous interventions, such as modifications of the diet (Table 4), are promising for the treatment of cognitive disorders.

To confirm this, another recent RCT [51] investigated the effect of a 1-year treatment with a Mediterranean-like diet (a well-known anti-inflammatory dietary approach) on cognitive function and fatigue in 72 MS patients. This study showed that the higher the adherence to the Mediterranean diet, the lower the risk of fatigue. No significant improvement was observed in relation to cognition, probably due to the small numbers of patients, therefore with the necessity for more robust clinical trials and longitudinal studies.

Another important aspect of the daily life that we have to consider is the maintenance of adequate sleep quality and quantity, according to a previous excellent review on pharmacological and non-pharmacological treatments [52]. Sleep disorders are associated with more severe cognitive decline, and poor quality sleep can appear long before any cognitive symptoms in MS patients.

In conclusion, non-pharmacological treatment is an emerging but promising approach: for this reason, there are several limits. Firstly, most of the cited studies in our review are single RCTs or single observational studies and the examined techniques were not repeated in other studies.

Secondly, despite the number of studies performed in the last few years concerning the rehabilitation of CI, there is very limited knowledge regarding how these techniques may be applied. In fact, a standardized training protocol does not exist, and the intervention in the studies we have reported vary in terms of:

- Single session duration (from 20 min to 2 h);
- Entire protocol duration (between 4 and 24 weeks);
- Weekly frequency (2–5 times per week).

(for more details, see the box “Intervention” of Table 2, Table 3 and Table 4).

Similarly, an even wider number of tests and cognitive batteries were applied, with mixed results. The use of the same assessment measure is important, because the patients are subject to repeated evaluations over time and this would allow a longitudinal comparison of the intra-patient data, but also to make the results comparable with those obtained by other international research studies. For this reason, the minimal assessment of cognitive functions in the MS (MACFIMS) battery was chosen by a panel of experts, who established which was the most valuable assessment tool and then prepared its cross-cultural validation. This battery is composed of seven tests, most of them utilized in the studies we have examined: the PASAT, SDMT, CVLT-II, BVMT-R, D-KEFS, Judgment of line orientation test (BJLO) and COWAT.

The aforementioned heterogeneity and the consequent limitations are consistent with those reported in a previous review [53], which explored a period earlier than the one we considered in our study, suggesting the possible presence of an intrinsic difficulty in the standardization of cognitive rehabilitation.

Therefore, future studies about the effects of non-pharmacological treatments on cognitive disorders have to face various challenges, both in the assessment and in the intervention. Further research is needed to identify the appropriate timing, dosing, frequency, setting and specificity of the treatment, but also to examine the feasibility and the reliability of the MACFIMS battery in highlighting the differences in cognitive performances after the intervention. Moreover, only a few studies present in the literature have evaluated the temporal stability of the interventions, so further studies should employ a longitudinal design to investigate how long the cognitive benefits last.

## Figures and Tables

**Table 1:** Type of non-pharmacological treatments of CI.

Cognitive Rehabilitation (CR)	
**Single-domain treatment (i.e., memory)**	Traditional CR [8,9] Adaptive n-back working memory training [10] Mental Visual Imagery (MVI; [11])
**Multi-domain treatment**	Program Cognitif pour Sclérose En Plaques French (ProCog-SEP program; [12]) Modified Story Memory Technique (mSMT; [13]) Strategy-based Techniques to Enhance Memory (STEM; [14]) Self-generation learning program (self-GEN trial; [15]) Adaptive program for cognitive and emotional deficits (ADACOG program; [16]) Additive interventions [17] Cognitive Occupation-Based Programme for People with Multiple Sclerosis (COB-MS; [18]) Traditional CR/Integrative approach: CR + neurologic music therapy [19]
**Group cognitive rehabilitation (GCR)**	[20,21]
**Computerized Cognitive Rehabilitation (CCR)**	NOROSOFT Mental Exercise Program [22] Memory, Attention, Problem Solving Skills in MS (MAPSS-MS; [23]) Cogmed Working Memory Training (CWMT; [24]) ERICA [25] COGNI-Track [26] Adaptive cognitive remediation (ACR; [27])/BrainHQ [28,29] RehaCom [30] Videogames (VG)/VG-like protocol [31,32] Virtual Reality (VR)/semi-immersive VR rehabilitation protocol [33,34]
**Exercise Training (ET)/** **Physical Activity (PA)**	ET: routine cycle ergometry training [35]; gait-training program with a Robotic Exoskeleton [36]; progressive aerobic exercise [37] PA: combined aerobic and Pilates [38]; “Start-to-Run” Program [39]; WalkWithMe application [40]
**Non-Invasive Brain Stimulation (NIBS)** **Brain Modulation**	Transcranial Direct Current Stimulation (tDCS; [41]) Remotely supervised-tDCS (RS-tDCS; [42]) EEG neurofeedback training [43]
**Miscellaneous Interventions**	Food supplement: GranaGard [44]

**Table 2:** In-presence cognitive and motor rehabilitative treatments.

Authors (Year)	Sample Size	Study Design	Intervention (Technique/Treatment and Duration)	Cognitive Results	MRI Results
Mousavi et al. (2018) [8]	60 patients (20 exp; 20 placebo; 20 controls)	RCT	Memory rehabilitation (one-hour sessions on a weekly basis for 8 weeks)	Improvement in working memory	
Arsoy et al. (2018) [22]	21 BMS patients; 22 non-BMS patients; 38 controls	RCT	Computer-assisted cognitive rehabilitation (NOROSOFT Mental Exercise Program; 5 days a week for 50 min)	Improvements in sustained attention, information processing speed, verbal fluency, categorical reasoning and executive functions	
Barry et al. (2018) [35]	19 patients (9 cases; 10 controls)	RCT	PA (cycled for 30 min at 65–75% age-predicted maximal heart rate, twice a week for 8 weeks)	Improvement in attention, executive function/cognitive flexibility and visuospatial memory (via CANTAB battery)	
Ernst et al. (2018) [11]	20 patients (10 cases; 10 controls)	RCT	MVI program (six two-hour individual sessions, one or twice per week)	Improvement in autobiographical memory	Enhanced neural activity in the left medial frontal regions and the right thalamus, in the left middle and inferior frontal gyrus, the left fusiform gyrus and left cerebellum
Goverover et al. (2018) [15]	35 patients (19 treatment; 16 placebo)	RCT	Self-generation learning program (self-GEN trial; six 60 min)	Improved learning and memory, self-regulation, and metacognition (Contextual memory test, memory for intentions test)	
Mani et al. (2018) [20]	34 patients	RCT	GCR (eight 2-h sessions of comprehensive group CR in 4 week)	Improvement in memory and executive function (ACE test, MFQ, WMS-R, WCST and BRIEF-A). No difference in attention (tested with CPT)	
Mousavi et al. (2020) [9]	60 patients	RCT	Compensatory strategies, internal and external memory aids, mnemonics, mental reviews and error-free learning (1-h sessions on a weekly basis for 8 weeks)	Increase of the everyday memory with short duration during FU (<5 weeks)	
Reilly et al. (2018) [18]	12 patients	Longitudinal study	COB-MS (eight sessions over 9 weeks, 60 min each session)	Improvements in verbal memory (CVLT-II), visual memory (BVMT-R), divided attention (TMT part B) and EMQ-R	
Androwis et al. (2019) [36]	4 patients (2 cases; 2 controls)	RCT	RE-gait training (case)/CGT (control) (8-session; 1 h/session)	Improvement in the processing speed (SDMT)	
Pineau et al. (2019) [16]	45 patients (24 cases; 21 controls)	Case- control Study	ADACOG: psycho-educational program focusing on cognitive and emotional dysfunctions (3 modules; each module 2 h every two weeks)	Less subjective self-reported cognitive deficits with MSNQ	
Brissart et al. (2020) [12]	128 patients (64 cases; 64 controls)	RCT	CR program (ProCog-SEP) in group (13 two-hour sessions over six months)	Improvement in learning index (Selective Reminding Test) and verbal and working memory (Digit span backward and Working memory, TAP)	
Chiaravalloti et al. (2020) [13]	30 patients (15 cases; 15 controls)	RCT	mSMT (10 sessions of the mSMT 2×/week for 5 weeks; sessions lasting 45–60 min)	Significant improvements in learning (both objective and self-reported), CVLT-II, SDMT	
Darestani et al. (2020) [30]	60 patients (30 cases; 30 controls)	RCT	Rehacom (10 sessions during 5 weeks—2 sessions per week and each session was 1 h)	Improved verbal performance with COWAT and CVLT-II	
Impellizzeri et al. (2020) [19]	30 patients (15 cases; 15 controls)	RCT	CR (controls: 6 times/week for 8 weeks) CR + NMT (cases: 3 times CR + 3 times NMT a week for 8 weeks)	Improvements in the BRB-N: selective reminding test long term storage, long term retrieval and delayed recall	
Lincoln et al. (2020) [21]	449 patients (205 cases; 204 controls)	RCT	GCR (delivered weekly to 4–6 participants for 10 weeks)	Small improvement on EMQ at both 6 and 12 months (subjective participant and relative reports of memory problems)	
Ozdogar et al. (2020) [31]	60 patients (21 VG; 19 conventional CR; 20 controls)	RCT	CCR (VG) (once a week for 8 weeks)	Improvement in nine-hole peg test (VG and conv. rehab); in VG: CVLT, SDMT and BVMT-R	
Ozkul et al. (2020) [38]	34 patients (17 cases; 17 controls)	RCT	PA (combined aerobic and Pilates; three sessions per week for 8 weeks)	Improvements in long-term verbal memory, visuospatial memory, verbal fluency, information processing speed	
Stimmel et al. (2020) [17]	30 patients (16 cases; 14 controls)	RCT	Additive interventions (in-person feedback and care-coordinator) phone calls	No significant difference pre- and post-intervention	
Chiaravalloti et al. (2021) [14]	20 patients (9 cases; 11 controls)	RCT	Memory Rehab via STEM protocol (Self-generation, spaced learning, and retrieval practice) (8 sessions: 2 sessions/week for 4 weeks; 30–45 min long)	Medium-large effect size on the CVLT-II total learning score	
De Luca et al. (2021) [25]	40 patients (20 cases; 20 controls)	RCT	CCR (ERICA software; 3 times a week for 8 weeks 45 min each session)	Improvements in memory, attention, and processing speed (test trough Montreal cognitive assessment, SDMT, SRT-LTS and SRT-D	
Gholami et al. (2021) [41]	24 patients (12 cases; 12 controls)	RCT	tDCS (8 consecutive daily tDCS sessions over the left DLPFC)	Improvement in reasoning and executive functions (assessed via CBS-CP, RBANS)	
Langeskov- Christensen et al. (2021) [37]	86 patients (43 cases; 43 controls)	RCT	PA (24-weeks progressive aerobic exercise)	Improvement in the SDMT	
Leonardi et al. (2021) [33]	30 patients (15 conventional CR; 15 VR)	RCT	VR (3 times a week for 8 weeks, each session lasting about 45 Min)	Improvement in learning ability, short-term verbal memory and lexical access ability for the VR group	
Turtola et al. (2021) [10]	24 patients (12 cases; 12 controls)	Case- control Study	Adaptive working memory training (20 sessions, each session 25–30 min, recommended rate of 5 sessions/week)	Enhancement of attention and cognitive control on untrained tasks	Potential limitations in the neural plasticity induced by working memory training
Maggio et al. (2022) [34]	60 patients (30 cases; 30 controls)	RCT	Semi-immersive VR training (three sessions/week, each session 60 min, for 8 weeks)	Improvements in visual perception, visuospatial abilities, short term visual memory working memory and executive functions, speed of information processing and sustained attention (PASAT 2′)	

**ACE:** Addenbrooke’s Cognitive Examination; **ADACOG:** Adaptive program for Cognitive and emotional deficits; **BMS:** Benign Multiple Sclerosis; **BRB-N:** Brief Repeatable Battery of Neuropsychological test; **BRIEF-A:** Behavior Rating Inventory of Executive Function-Adult; **BVMT-R:** Brief Visuospatial Memory Test-Revised; **CANTAB:** Cambridge Neuropsychological Test Automated Battery; **CBS-CP:** Cambridge Brain Sciences-Cognitive Platform; **CCR:** Computerized Cognitive Rehabilitation; **CGT:** Conventional Gait Therapy; **COB-MS:** Cognitive Occupation-Based program for people with Multiple Sclerosis; **COWAT:** Controlled Oral Word Association Test; **CPT:** Continuous Performance Test; **CR:** Cognitive Rehabilitation; **CVLT:** California Verbal Learning Test; **CVLT-II:** California Verbal Learning Test—second edition; **DLPFC:** Dorsolateral-prefrontal Cortex; **EMQ:** Everyday Memory Questionnaire; **EMQ-R:** Everyday Memory Questionnaire-Revised; **FU:** Follow-Up; **GCR:** Group Cognitive Rehabilitation; **MFQ:** Memory Functioning Questionnaire; **MRI:** Magnetic Resonance Imaging; **MS:** Multiple Sclerosis; **MSNQ:** Multiple Sclerosis Neuropsychological Screening Questionnaire; **MVI:** Mental Visual Imagery; **mSMT:** Modified Story Memory Technique; **NMT:** Neurologic Music Therapy; **non-BMS:** non-Benign Multiple Sclerosis; **PA:** Physical Activity; **PASAT:** Paced Auditory Serial Addition Task; **PASAT 2′:** 2 second-version PASAT; **PASAT 3′:** 3 second-version PASAT; **ProCog-SEP:** Program Cognitif pour Sclérose En Plaques; **RBANS:** Repeatable Battery for the Assessment of Neuropsychological Status; **RCT:** Randomized Controlled Trial; **RE:** Robotic Exoskeleton; **SDMT:** Symbol Digit Modalities Test; **SRT-D:** Selective Reminding Test—Delayed recall of the selective reminding test; **SRT-LTS:** Selective Reminding Test-Long Term Storage; **STEM:** Strategy-based Training to Enhance Memory; **TAP:** Test of Attentional Performance; **tDCS:** Transcranial Direct Current Stimulation; **VG:** Video Games; **VR:** Virtual Reality; **WCST:** Wisconsin Card Sorting Test; **WMS-R:** Wechsler Memory Scale-Revised.

**Table 3:** Cognitive and Motor Tele-Rehabilitation.

Authors (Year)	Sample Size	Study Design	Intervention (Technique/Treatment and Duration)	Cognitive Results	MRI Results
Charvet et al. (2017) [27]	135 patients (74 cases; 61 controls)	RCT	Online ACR program (research version of BrainHQ program) (1 h/day, 5 days/week over 12 weeks)	Improvement in Processing Speed (PASAT) and Visual Scanning (D-KEFS) in active group	
Charvet et al. (2018) [42]	45 patients (25 cases; 20 controls)	RCT	RS-tDCS + CT (ten 20-min sessions of tDCS paired with a CT program); CT only condition (ten 20-min sessions of training)	Improvement in complex attention and response variability composites (compared to the only CT group)	
Stuifbergen et al. (2018) [23]	183 patients (93 cases; 90 controls)	RCT	CCR (MAPSS-MS intervention: 3 daily sessions of 45 min, three times per week; 8 weeks)	Improvement in the CVLT Delayed Score, PASAT 3′, COWAT and PROMIS Cognitive Abilities scale	
Feys et al. (2019) [39]	42 patients (21 cases; 21 controls)	RCT	PA: Individualized training in preparation of a running event (3 times weekly according to a personalized training intensity schedule; 12 weeks)	No important differences after training except for SPART	
Fuchs et al. (2019) [28]	51 patients	Exploratory Study	BrainHQ (online restorative cognitive training program: 1 training session/day 45–60 min, for 5 days each week)	Improvement in SDMT	
Bonzano et al. (2020) [26]	36 patients (18 cases; 18 controls)	Longitudinal Study	Working memory training: COGNI-TRAcK (8-week training; five 30-min sessions a week)	Improvement in PASAT	Brain activation map during PVSAT more similar to healthy participants after treatment (clusters mainly located in the right cerebellum and in the left hemisphere: precuneus and superior parietal lobule, precentral and superior frontal gyri)
Van Geel et al. (2020) [40]	19 patients	Longitudinal study	PA (WalkWithMe, a personalized mobile application that helps to walk at home; 10 weeks)	Improvements in SDMT and PASAT	
Vilou et al. (2020) [29]	47 RRMS patients (23 cases; 24 controls)	Explorative Study	BrainHQ web-based platform (20 min each session; 6 weeks)	Improvements in BVMT-R, GVLT, TMT-A and BICAMS (memory). No statistically significant difference in SDMT	
Blair et al. (2021) [24]	30 patients (15 cases; 15 controls)	RCT	CCR with CWMT (25 training sessions; 8 exercises daily, approximately 30–45 min)	Improvements in D-KEFS Color-Word Interference Test, Letter-Number Sequencing and Digit Span	
Bove et al. (2021) [32]	44 patients (23 cases; 21 controls)	RCT	Tablet-based VG-like digital treatment (25 min/day, 5 days/week, for 6 weeks)	Improvement in SDMT	
Pinter et al. (2021) [43]	14 patients	Longitudinal Study	EEG-based neurofeedback via tele-rehabilitation (10 training sessions within 3–4 weeks)		Increased microstructural WM integrity in the left corticospinal tract, left anterior thalamic radiation and increased functional connectivity of salience network

**ACR:** adaptive cognitive remediation; **BICAMS:** Brief International Cognitive Assessment for Multiple Sclerosis; **BVMT-R:** Brief Visuospatial Memory Test-Revised; **CCR:** Computerized Cognitive Rehabilitation; **COGNI-TRAcK:** Cognitive Training Kit; **CVLT:** California Verbal Learning Test; **COWAT:** Controlled Oral Word Association Test; **CWMT:** Cogmed Working Memory Training; **CT:** Cognitive Training; **D-KEFS:** Delis–Kaplan Executive Function System; **EEG:** Electroencephalogram; **GVLT:** Greek Verbal Learning Test; **MAPSS-MS:** Memory, Attention, Problem Solving Skills in MS; **MRI:** Magnetic Resonance Imaging; **PA:** Physical Activity; **PASAT:** Paced Auditory Serial Addition Task; **PASAT 3′:** 3 second-version PASAT; **PROMIS:** Patient Reported Outcomes Measurement Information System; **PVSAT:** Paced Visual Serial Addition Test; **RCT:** Randomized Controlled Trial; **RRMS:** Relapsing-Remittent Multiple Sclerosis; **RS-tDCS:** Remotely-Supervised Transcranial Direct Current Stimulation; **SDMT:** Symbol Digit Modalities Test; **SPART:** Spatial Recall Test; **TMT-A:** Trail Making Test—Part A; **WM:** White Matter.

**Table 4:** Miscellaneous.

Authors (Year)	Sample Size	Study Design	Intervention (Technique/Treatment and Duration)	Cognitive Results	MRI Results
Petrou et al. (2021) [44]	30 patients (15 cases; 15 controls)	RCT	Diet (GranaGard, food supplement consisting in a self-emulsion nano formulation of pomegranate seed oil)	Improvement in CVLT-II, no change in SDMT	

**CVLT-II:** California Verbal Learning Test—second edition; **MRI:** Magnetic Resonance Imaging; **RCT:** Randomized Controlled Trial; **SDMT:** Symbol Digit Modalities Test.

## Data Availability

Not applicable.
